# The Use of NS1 Rapid Diagnostic Test and qRT-PCR to Complement IgM ELISA for Improved Dengue Diagnosis from Single Specimen

**DOI:** 10.1038/srep27663

**Published:** 2016-06-09

**Authors:** Boon-Teong Teoh, Sing-Sin Sam, Kim-Kee Tan, Jefree Johari, Juraina Abd-Jamil, Poh-Sim Hooi, Sazaly AbuBakar

**Affiliations:** 1 Tropical Infectious Diseases Research and Education Centre (TIDREC), Department of Medical Microbiology, Faculty of Medicine, University of Malaya, Kuala Lumpur, Malaysia

## Abstract

Timely and accurate dengue diagnosis is important for differential diagnosis and immediate implementation of appropriate disease control measures. In this study, we compared the usefulness and applicability of NS1 RDT (NS1 Ag Strip) and qRT-PCR tests in complementing the IgM ELISA for dengue diagnosis on single serum specimen (n = 375). The NS1 Ag Strip and qRT-PCR showed a fair concordance (κ = 0.207, *p* = 0.001). While the NS1 Ag Strip showed higher positivity than qRT-PCR for acute (97.8% vs. 84.8%) and post-acute samples (94.8% vs. 71.8%) of primary infection, qRT-PCR showed higher positivity for acute (58.1% vs. 48.4%) and post-acute (50.0% vs.41.4%) samples in secondary infection. IgM ELISA showed higher positivity in samples from secondary dengue (74.2–94.8%) than in those from primary dengue (21.7–64.1%). More primary dengue samples showed positive with combined NS1 Ag Strip/IgM ELISA (99.0% vs. 92.8%) whereas more secondary samples showed positive with combined qRT-PCR/IgM ELISA (99.4% vs. 96.2%). Combined NS1 Ag Strip/IgM ELISA is a suitable combination tests for timely and accurate dengue diagnosis on single serum specimen. If complemented with qRT-PCR, combined NS1 Ag Strip/IgM ELISA would improve detection of secondary dengue samples.

Dengue is among the most important mosquito-borne viral disease worldwide^1^. Up to 3.97 billion people living in 128 countries are now at risk of contracting dengue^2^, with an estimated 390 million of new infections annually^3^. The disease spectrum ranges from mild dengue fever (DF) to severe and deadly dengue hemorrhagic fever (DHF) and dengue shock syndrome (DSS)^4^. All four dengue virus (DENV) serotypes (DENV-1, DENV-2, DENV-3 and DENV-4) can cause dengue^5^,^6^. The virus is transmitted following mosquito bites of febrile patients in viremic phase of infection, which is usually during the first 3–4 days of illness^7^. In the absence of a widely implemented vaccination^8^ or approved antiviral therapy^9^,^10^ against dengue, vector control and personal protective measures against mosquito bites remain the only ways to reduce the burden of dengue. Immediate implementation of appropriate infected mosquito control measures, however, requires a timely and accurate laboratory diagnosis for prompt identification of dengue cases^11^. Early detection of dengue is also important for better clinical management of patients to prevent unnecessary intervention measures such as antibiotic prescriptions and unnecessary hospitalization.

Timely laboratory diagnosis of dengue relies on the detection of DENV RNA, non-structural protein 1 (NS1) or anti-DENV IgM in a single serum specimen obtained during the patient’s first visit to hospital or clinic. As the sensitivities of the different tests could depend on the time of specimen collection, whether during the viremic phase or post-viremic phase, a combination of multiple tests is needed to achieve accurate diagnosis. In general, serological diagnosis of dengue by IgM-capture enzyme-linked immunosorbent assay (ELISA) is the most widely used approach^12^,^13^,^14^. It has been the backbone of dengue diagnosis in many public healthcare centers due to its usefulness especially when the majority (>80%) of the dengue patients had illness beyond four days upon presentation for medical attention, that the patients were most likely already have detectable IgM antibodies^15^. While serology showed limitations for diagnosing dengue in samples collected before seroconversion, DENV NS1 antigen detection^16^,^17^ or DENV RNA detection techniques such as reverse transcription-polymerase chain reaction (RT-PCR)^18^,^19^ and real-time quantitative RT-PCR (qRT-PCR)^20^,^21^ are useful for serum specimens obtained within the acute phase of illness. The DENV NS1 protein in serum sample is detectable either by a lateral flow rapid diagnostic test (RDT) or ELISA format^12^ as early as the onset of fever until 8–9 days later, a post-viremic time when the nucleic acid detection has often become negative^22^,^23^,^24^. Despite DENV NS1 test offering a longer detection window than DENV RNA, a decreased diagnostic sensitivity of NS1 detection in secondary dengue samples has been reported^25^,^26^.

In the present study, we compared the usefulness and applicability of the NS1 RDT in complementing the IgM ELISA test for the detection of DENV infection and the value of nucleic acid tests using single serum specimen obtained during dengue-suspected patient’s first visit to hospital in endemic dengue setting.

## Results

**Figure 1:**
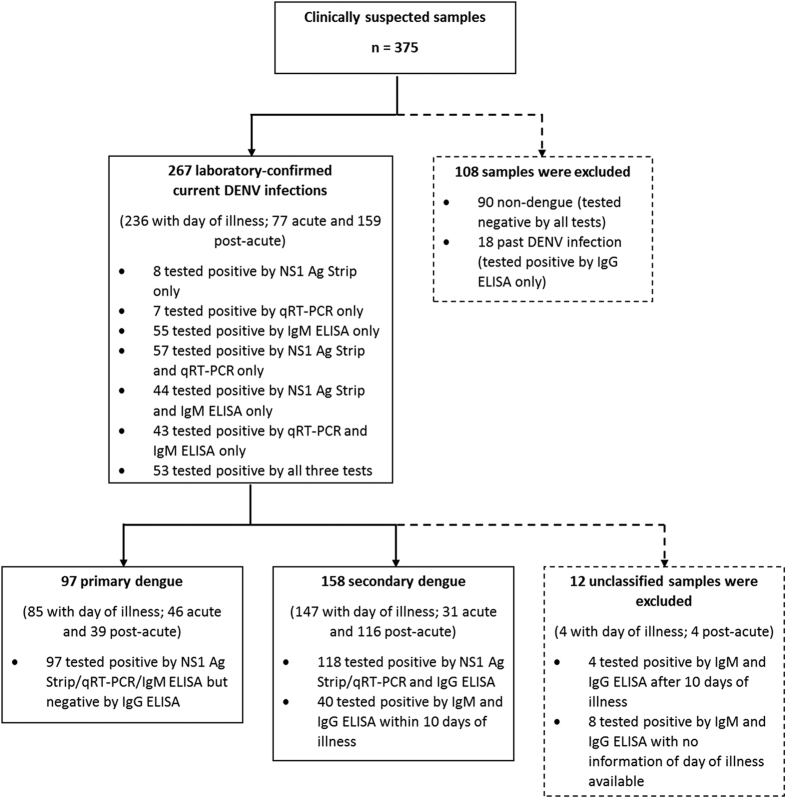
Flow chart of selection and exclusion of samples for analyses.

In this study, 375 serum samples obtained from clinically dengue-suspected patients were tested using the NS1 Ag Strip, qRT-PCR and SD Dengue IgM- and IgG-Capture ELISA. Out of the 375 samples, current DENV infection was confirmed in 267 samples (71.2%) by qRT-PCR, NS1 Ag Strip and/or dengue IgM ELISA (Table 1, Fig. 1). Of the 267 samples, DENV RNA and NS1 antigen were detected in 160 (59.9%) and 162 (60.7%) samples, respectively. Conversely, 195 of the 267 (73.0%) samples tested positive by dengue IgM ELISA. Eighteen samples were identified as from those who had past DENV infections, as only their dengue IgG tested positive.

**Figure 2:**
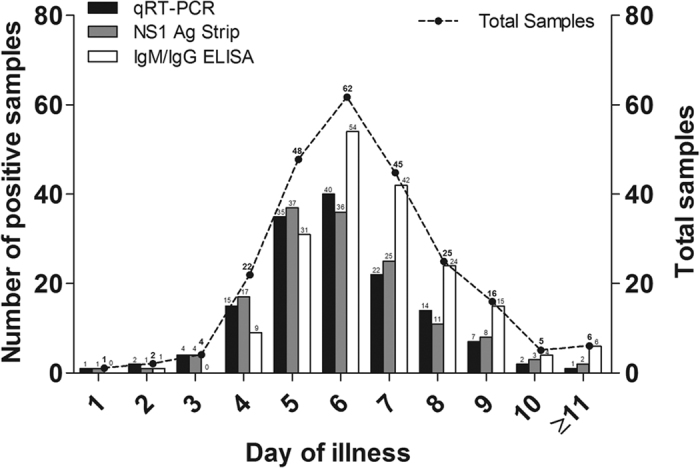
Comparison of the performance of various dengue diagnostic methods against laboratory-confirmed dengue samples according to the day of illness.

The performance of all of the diagnostic methods was analyzed based on the day of illness when the samples were obtained (Fig. 2). The date of illness onset was available for 236 (out of 267) dengue-confirmed cases and the samples ranged from day 1 to ≥day 11 of illness (median = day 6.0). Eighty-six percent (85.6%, 202/236) of the samples were obtained between day 4 and day 8 of illness. Of 236 samples, 77 were acute samples (≤day 5) while 159 were post-acute samples (>day 5). The IgM/IgG ELISA tested positive in 53.2% (41/77) and 91.2% (145/159) of the acute and post-acute samples, respectively. In contrast, the NS1 Ag Strip and qRT-PCR assays tested positive in 74.0–77.9% (57–60 out of 77) of the acute samples and the positive detections decreased to 53.5–54.1% (85–86 out of 159) for the post-acute samples. Both the NS1 Ag Strip and qRT-PCR assays were used for early detection of the DENV infection. These two assays, however, showed a fair concordance with kappa value of 0.207 (*p* = 0.001).

Out of the 267 dengue-confirmed cases, 97 and 158 cases were classified as primary and secondary dengue, respectively, using the criteria as mentioned in the Methods section (Fig. 1). The day of illness was available for 85 primary dengue cases and 147 secondary dengue cases. There were more acute samples from the primary dengue cases (54.1%; 46 of 85) than those from secondary dengue cases (21.1%; 31 of 147), whereas the secondary cases had more post-acute samples (78.9%; 116 of 147) in comparison to the primary cases (45.9%; 39 of 85).

**Figure 3:**
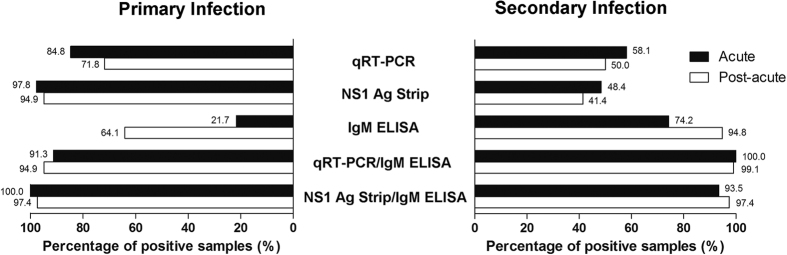
Comparison of the performance of qRT-PCR, NS1 Ag Strip and IgM ELISA assays against acute and post-acute dengue samples in primary and secondary DENV infection. The test sensitivities between primary and secondary infection were compared using Chi-square test or Fisher’s exact test (two-tailed). Significant differences (*p* < 0.01) of test sensitivities between primary and secondary infection were obtained for all tests.

A segregation analysis based on the primary and secondary DENV infections was performed (Fig. 3). The NS1 Ag Strip test showed higher positivity than qRT-PCR for both acute (97.8% vs. 84.8%) and post-acute samples (94.8% vs. 71.8%) from the primary infection cases. However, among the secondary infection cases, qRT-PCR showed higher positivity than NS1 Ag Strip for both acute (58.1% vs. 48.4%) and post-acute (50.0% vs.41.4%) samples. Kappa values of 0.485 (*p* < 0.001) and −0.022 (*p* = 0.776) were obtained when comparing between the performance of NS1 and qRT-PCR among samples of primary and secondary dengue, respectively. Seventy-nine percent (79.0%; 37/47) of the samples that tested positive by NS1 Ag Strip but negative by qRT-PCR were samples obtained after day 5 of the illness onset. On the other hand, 98.0% (49/50) of the samples that tested negative by NS1 Ag Strip but positive by qRT-PCR were samples from secondary dengue cases. Both the NS1 Ag Strip and qRT-PCR tests showed significant decrease (*p* < 0.001) in positive detection in samples of secondary dengue cases in comparison to those of primary dengue cases. On the other hand, IgM ELISA showed significant (p < 0.001) higher positivity in both the acute samples (74.2% vs 21.7%) and post-acute samples (94.8% vs 64.1%) from secondary dengue than in those from primary dengue. In comparison to the combined qRT-PCR/IgM ELISA, the combined NS1 Ag Strip/IgM ELISA showed higher sensitivity (97.4–100.0% vs 91.3–94.9%) for both acute and post-acute samples in primary dengue. Whereas the combined qRT-PCR/IgM ELISA showed higher sensitivity (99.1–100.0% vs 93.5–97.4%) than that of combined NS1 Ag Strip/IgM ELISA for both acute and post-acute samples in secondary dengue.

**Figure 4:**
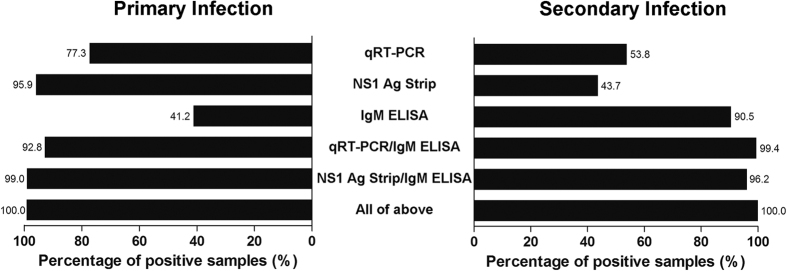
Sensitivity of various dengue diagnostic methods against laboratory-confirmed dengue samples in primary and secondary DENV infection. The test sensitivities among groups were compared using McNemar’s exact test (two-tailed). The sensitivities of combined NS1 Ag Strip/IgM ELISA and combined qRT-PCR/IgM ELISA tests were significant higher (*p* < 0.01) than those of individual tests.

In primary dengue, the combined NS1 Ag Strip/IgM ELISA tests detected 99.0% of the samples, while the combined qRT-PCR/IgM ELISA tests detected 92.8% of the samples (Fig. 4). In contrast, the combined qRT-PCR/IgM ELISA tests showed higher positivity (99.4%) in samples of secondary dengue in comparison to that of the combined NS1 Ag Strip/IgM ELISA tests (96.2%). Both combined NS1 Ag Strip/IgM ELISA tests and combined qRT-PCR/IgM ELISA tests showed significant increases (*p* < 0.01) in positive detections in comparison to the use of IgM ELISA alone, which had positive detections of only 41.2% of primary dengue samples and 90.5% of secondary dengue samples.

## Discussion

In the present study, the usefulness and applicability of the NS1 RDT and qRT-PCR in complementing the IgM ELISA test were assessed for possible accurate and timely detection of DENV infection in single serum specimen during patient’s first visit. Stratified analysis based on the immune status was performed to assess the test performance in primary compared with secondary infection. The findings showed that the NS1 RDT using NS1 Ag Strip was more sensitive than the qRT-PCR for detection of both acute and post-acute samples from primary DENV infection. In contrast, the qRT-PCR showed higher sensitivity than the NS1 Ag Strip for the detection of both acute and post-acute samples from secondary DENV infection. The combined NS1 Ag Strip/IgM ELISA tests showed higher sensitivity than the combined qRT-PCR/IgM ELISA tests for the detection of primary dengue, whereas the combined qRT-PCR/IgM ELISA tests showed higher sensitivity than the combined NS1 Ag Strip/IgM ELISA tests for the detection of secondary dengue.

The diagnostic performance of NS1 RDT has been evaluated and reported in numerous studies^12^,^26^,^27^,^28^,^29^,^30^. In agreement with most of the previous findings, we observed a high diagnostic sensitivity of NS1 Ag Strip test for detection of primary dengue cases. A longer duration of NS1 antigenemia than that of viremia in primary DENV infection makes NS1 detection method an advantage over DENV nucleic acid detection technique for dengue diagnosis during acute phase of infection^22^,^23^,^24^. In secondary DENV infection, however, a decreased sensitivity of NS1 Ag Strip test was observed in our study similar to earlier findings^25^,^26^. Anamnestic secondary anti-NS1 IgG response generated during the acute stage of infection could mask the epitopes of NS1 protein via the formation of IgG-NS1 immune complex, leading to shorten duration of NS1 antigenemia^31^. The masked epitopes of NS1 protein would not be detected by NS1 diagnostic test unless an acid or heat treatment is performed to dissociate the NS1 protein from the IgG antibodies before the diagnosis^31^,^32^. This dissociation step, however, may not be practically useful in routine diagnostic laboratories.

Similar to the NS1 Ag Strip test, qRT-PCR showed decreased sensitivity in detecting the secondary dengue in comparison to the primary dengue. This could be attributed to earlier and faster clearance of viruses by the pre-existing memory antibodies^22^,^24^. In this study, the mean viral load was generally lower in secondary dengue than that in primary dengue (see Supplementary Figure). Nevertheless, in some cases, although the DENV would form virion-antibody complexes during the acute stage of secondary infection, the DENV RNA could still be extracted by any RNA extraction methods. Therefore, qRT-PCR still showed greater sensitivity than that of NS1 Ag Strip test in secondary dengue samples, despite the high proportion of post-acute secondary dengue samples in our study. A recent external quality assessment (EQA) of dengue diagnostics in World Health Organization (WHO) Western Pacific Region revealed that only less than 50% of the national-level public health laboratories participated perform DENV NS1 antigen detection as part of their routine diagnostics; majority of the laboratories performed viral nucleic acid detection to complement the IgM ELISA test^14^. This observation could be attributed to the fact that DENV NS1 detection test has been reported to have lower sensitivity in detecting secondary infection which is common in dengue endemic regions.

In this study, we showed that the diagnostic performance of IgM ELISA was found to be better for the detection of secondary dengue than primary dengue for both acute and post-acute samples. Early appearance of anti-DENV IgM antibody in secondary infection, as compared to primary infection, has also been reported in an earlier study and this could have contributed to the improved sensitivity of IgM ELISA test in detecting secondary dengue samples^33^. While anti-dengue IgM ELISA showed low positive detection among the primary dengue samples (41.2%), the use of NS1 Ag Strip or qRT-PCR as complementary test greatly improved the sensitivity of detection (92.6–99.0%) in these samples. These findings were in agreement with those of previous studies^29^,^30^,^34^. On the other hand, the present study was one of the first to perform comprehensive comparison between the sensitivity of the combined NS1 Ag Strip/IgM ELISA and the sensitivity of the combined qRT-PCR/IgM ELISA assays in samples stratified according to the dengue immune status and day of illness. In general, both combined NS1 Ag Strip/IgM ELISA and combined qRT-PCR/IgM ELISA tests showed comparable diagnostic sensitivities in both primary and secondary dengue, though the combined NS1 Ag Strip/IgM ELISA showed slightly higher sensitivity (99% vs 93%) in primary dengue while the combined qRT-PCR/IgM ELISA showed slightly higher sensitivity (99% vs 96%) in secondary dengue. Nevertheless, the NS1 Ag Strip is a preferable complementary test over the qRT-PCR due to its ease of use, rapid run time (<30 minutes) and minimal equipment features. These advantages also made the NS1 Ag Strip, rather than the qRT-PCR, an attractive test to be used for dengue diagnosis in rural healthcare facilities or clinics where resource is limited.

The NS1 Ag Strip test alone yields similar sensitivity as that of combined NS1/IgM tests for samples of primary infection. In view of cost effectiveness, the NS1 strip test alone might be sufficient for dengue diagnosis in the dengue non-endemic regions where primary infection is prevalent. A negative detection of NS1 (~4%), however, should be interpreted with care. Subjecting the NS1-negative samples for the detection of anti-DENV IgM could help to prevent the reporting of false negative results, even though the IgM ELISA test has a relatively slow turnaround time. Without the confirmation by complementary test, the clinical judgment for rapid treatment or triaging of patients is possible but can be difficult especially due to the overlapping of disease presentations of dengue with the other viral or bacterial infections. It has been reported previously that the misdiagnosis of dengue by NS1 test, most likely in secondary infection, can impede proper treatment of dengue patients where the febrile patients were given antibiotics instead^35^. This emphasized that the complementary tests are necessary to improve the laboratory diagnosis of dengue in these cases and thus enable more accurate and proper treatment.

On the other hand, the combined NS1 Ag Strip/IgM ELISA test could work well in the dengue endemic regions where secondary infection is common, given that a good detection sensitivity (96.2%) was observed with an approximately 4% of misdetection in our secondary dengue cases. Nonetheless, it is crucial to diagnose these misdetections with a nucleic acid detection assay as secondary dengue is often associated with the development of severe dengue^36^. Recently, the isothermal nucleic acid amplification assays such as the RT-loop-mediated isothermal amplification (RT-LAMP) and RT-recombinase polymerase amplification (RT-RPA) have been established for the detection of DENV RNA in patient sera without the need of thermocycling^15^,^37^,^38^. The RT-LAMP and RT-RPA assays could be a promising alternative to qRT-PCR for the detection of DENV infection in resource limited settings as the tests do not require expensive equipment and high level of laboratory skills to perform.

There are, however, several limitations to our study. The difficulties in getting convalescent samples from patients after recovery have limited the application of hemagglutination inhibition (HI) assay for classification of patient’s dengue immune response^39^. Therefore, the classification of the patient immune status based on a single serum specimen was performed in this study. As been previously described, the classification of dengue immune response based on single acute-phase serum sample, either by the IgM/IgG ratio^40^,^41^,^42^, the IgG avidity^43^ or a two-dimensional classifier using just the IgG levels and days of symptoms^44^, has claimed good sensitivity and specificity. In our study, we made the classification not only based on the presence of IgM and IgG in a single serum specimen, but also based on the detection of DENV RNA or NS1, in relation to the day post-onset of illness. Twelve patients, however, were remained unclassified and excluded from the stratification analysis based on the dengue immune status. Other than immune status, the serotype of infecting DENV has been shown to influence the sensitivity of NS1 detection^27^,^45^. The information of infecting DENV serotype, however, was not available in this study to assess whether the diagnostic tests used were bias toward any virus serotype.

Our findings encourage the use of NS1 Ag Strip test to complement the anti-DENV IgM ELISA for accurate and timely detection of DENV infection using single serum specimen. The results of misdetections of the combined NS1 Ag Strip/IgM ELISA tests especially in dengue endemic regions, however, have to be cautiously interpreted. Complementing the combined NS1 Ag Strip/IgM ELISA tests with a sensitive nucleic acid-based test is important to rule out the false negative in dengue endemic regions where secondary dengue is common.

## Methods

### Ethics statement

The study was approved by the University Malaya Medical Center (UMMC) Medical Ethics Committee (Ethics Committee/IRB Reference Number: 1059.65). All experiments were performed in accordance with the approved guidelines. The serum samples were obtained without written consents from the patients as the samples were obtained retrospectively from UMMC Microbiology Laboratory Diagnostic Repository. The need for consent was waived by the UMMC Medical Ethics Committee.

### Clinical samples

Three hundred and seventy-five serum samples from 375 anonymous patients clinically suspected to be infected with DENV at the UMMC during the period from June to August 2013 were obtained for this study. The serum samples were collected during the patients’ first visit to the hospital. The serum samples were simultaneously tested for the presence of the DENV NS1 antigen, DENV RNA, and anti-DENV IgM and IgG antibodies. Records for the date of illness onset were provided by the UMMC Patient Information Department.

### Detection of DENV NS1 antigen

The DENV NS1 RDT used for the detection of DENV NS1 antigen was the Dengue NS1 Ag Strip (catalog number: 70700, BioRad, France). The NS1 Ag Strip was the most evaluated kit with the reported sensitivity and specificity ranged from 58.6–98.9% and 90.6–100%, respectively, using samples from Asian populations^46^. Tests were performed strictly following the manufacturer’s recommended protocol. One drop of migration buffer was added to 50 μl of the patient serum samples in a sample tube. The strip was then placed vertically in the tube. Results were read at 15 minutes and 30 minutes after adding the strip to the tube. The appearance of T and C lines indicated positive result. The appearance of C line alone indicated a negative result, whereas the absence of C line indicated an invalid result.

### Detection of DENV RNA

Total RNA was extracted from 140 μl of the patient serum samples using the QIAamp Viral RNA Mini Kit (Qiagen, Germany) according to the manufacturer’s protocol. The RNA was eluted in 60 μl of nuclease-free water and stored at −80 °C until needed. The extracted DENV RNA was detected and quantified using the Genesig real-time qRT-PCR DENV detection kit (PrimerDesign Ltd., U.K.) as previously described^15^,^37^. The DENV qRT-PCR from Genesig is developed and sold for research purposes only. It has been, however, evaluated in our previous study and has shown higher sensitivity in comparison to that of in-house qRT-PCR assay^47^. It has also been used as the reference assay in detection of DENV RNA in our laboratory for years.

### Detection of anti-DENV IgM and IgG antibodies

The presence of anti-DENV IgM and IgG antibodies were detected using the SD Dengue IgM- and IgG-Capture ELISA kits (Standard Diagnosis Inc., Korea), respectively, following the manufacturer’s recommended protocol. The IgM and IgG ELISA kits from SD have been used for the routine diagnostic practice for dengue in the UMMC. The ELISA tests were performed in the diagnostic laboratory of the UMMC.

### Interpretation of the diagnostic test results

Results for the NS1 Ag Strip, qRT-PCR, and ELISA were analyzed and compared. Current DENV infection was confirmed by the positive detection of DENV NS1 with NS1 Ag Strip, the positive detection of DENV RNA with qRT-PCR, or the presence of anti-DENV IgM detected with ELISA. Dengue-positive serum samples obtained within day 5 and after day 5 of illness were defined as acute and post-acute specimens, respectively. The serum sample was classified as a primary DENV infection when the sample tested positive by the NS1 Ag Strip, qRT-PCR or IgM ELISA in the absence of IgG. The serum sample was classified as a secondary DENV infection following two criteria: (i) the presence of anti-DENV IgG concurrent with the positive detection of the DENV RNA genome or NS1 antigen, (ii) the positive detection of both of the anti-DENV IgG and IgM in the serum sample obtained within 10 days after onset of illness^36^,^48^. The co-appearance of IgG and IgM after 10 days of illness, however, remained unclassified. When the information of day of illness was not available, the IgG and IgM-positive serum sample also remained unclassified. Serum samples tested positive only for anti-DENV IgG was identified as a reflection of past DENV infection.

### Statistical analysis

All of the statistical analyses were performed using IBM SPSS Statistics, version 21 (IBM Corporation, New York, United States). The degrees of agreement between the NS1 Ag Strip and qRT-PCR test results were determined with the kappa value (κ). A Chi-square test or Fisher’s exact test (two-tailed) was performed to compare the test performance between primary and secondary infection. McNemar’s exact test (two-tailed) was performed to compare the performance between combined diagnostic tests and individual test. In this study, a *p*-value of  < 0.01 was used to suggest significant results.

## Additional Information

**How to cite this article**: Teoh, B.-T. *et al.* The Use of NS1 Rapid Diagnostic Test and qRT-PCR to Complement IgM ELISA for Improved Dengue Diagnosis from Single Specimen. *Sci. Rep.* **6**, 27663; doi: 10.1038/srep27663 (2016).

## Supplementary Material

Supplementary Information

## Figures and Tables

**Table 1:** Summary of dengue detection in serum samples from clinically dengue-suspected patients at UMMC using qRT-PCR, NS1 Ag Strip, IgM and IgG ELISA.

Assays	Results^a^	IgM ELISA		IgG ELISA	
		Positive (n = 195)	Negative (n = 180)	Positive (n = 188^b^,^c^,^d^)	Negative (n = 187)
		n (%)	n (%)	n (%)	n (%)
qRT-PCR	Positive (n = 160)	96 (25.6)	64 (17.1)	85 (22.7)	75 (20.0)
	Negative (n = 215)	99 (26.4)	116 (30.9)	103 (27.5)	112 (29.8)
NS1 Ag Strip	Positive (n = 162)	97 (25.9)	65 (17.3)	69 (18.4)	93 (24.8)
	Negative (n = 213)	98 (26.1)	115 (30.7)	119 (31.7)	94 (25.1)

^a^Out of 375 sera, acute DENV infection was confirmed in 267 samples by qRT-PCR, NS1 Ag Strip and/or anti-DENV IgM ELISA.

^b^One hundred and eighteen anti-DENV IgG positive samples tested positive by qRT-PCR or NS1 Ag Strip.

^c^Forty-one anti-DENV IgG positive samples obtained within 10 days of illness tested positive by anti-DENV IgM ELISA alone.

^d^Eighteen anti-DENV IgG positive samples tested negative by anti-DENV IgM ELISA, NS1 Ag Strip and qRT-PCR.
