# Supplementary material for: The Use of NS1 Rapid Diagnostic Test and qRT-PCR to Complement IgM ELISA for Improved Dengue Diagnosis from Single Specimen
Source: Sci Rep. 2016 Jun 9;6:27663. doi: 10.1038/srep27663 (PMC4899743; doi:10.1038/srep27663)
Supplement: Supplementary Information [file srep27663-s1.pdf]

Title: The Use of NS1 Rapid Diagnostic Test and qRT-PCR to Complement IgM ELISA for Improved Dengue Diagnosis from Single Specimen

Boon-Teong Teoh, Sing-Sin Sam, Kim-Kee Tan, Jefree Johari, Juraina Abd-Jamil, Poh-Sim Hooi, and Sazaly AbuBakar*

Tropical Infectious Diseases Research and Education Centre (TIDREC), Department of Medical Microbiology, Faculty of Medicine, University of Malaya, Kuala Lumpur, Malaysia

*Corresponding author

Sazaly AbuBakar

Email: sazaly@um.edu.my

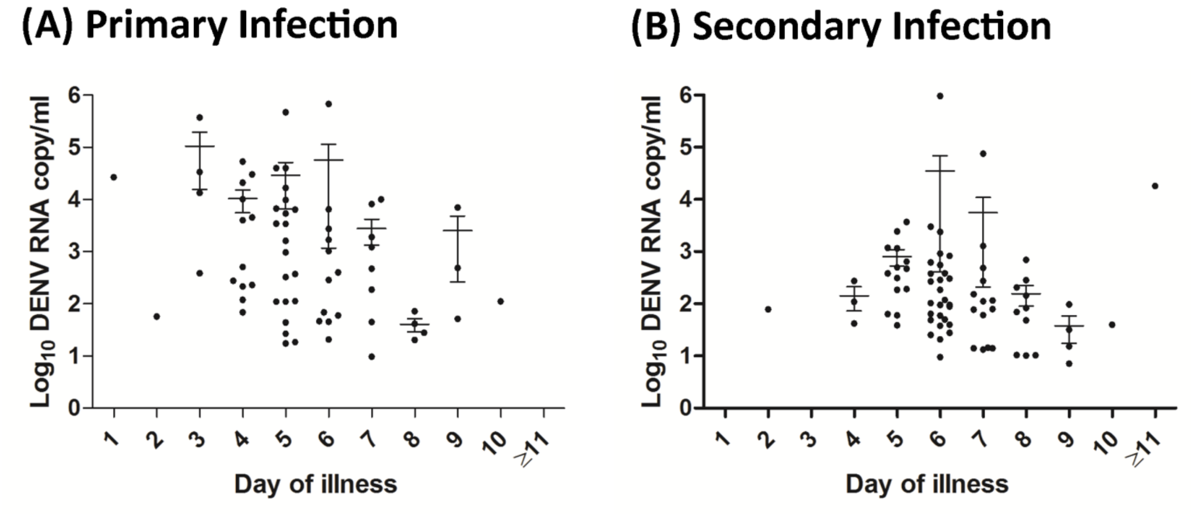

Supplementary Figure. Viral loads of dengue cases tested positive by qRT-PCR according to the day of illness. (A) Viral loads of the primary dengue cases (n = 67). (B) Viral loads of the secondary dengue cases (n = 76). The error bars indicate the standard errors of the viral loads from the mean.
